# A multitask framework based on CA-EfficientNetV2 for the prediction of glioma molecular biomarkers

**DOI:** 10.3389/fneur.2025.1609594

**Published:** 2025-07-18

**Authors:** Qian Xu, Feng Ning Liang, Ya Ru Cao, Jin Duan, Teng Cui, Teng Zhao, Hong Zhu

**Affiliations:** ^1^Department of Radiology, The Affiliated Hospital of Xuzhou Medical University, Xuzhou, Jiangsu, China; ^2^School of Medical Information and Engineering, Xuzhou Medical University, Xuzhou, Jiangsu, China; ^3^School of Medical Imaging, Xuzhou Medical University, Xuzhou, Jiangsu, China

**Keywords:** EfficientNetv2, glioma, IDH, MGMT, vision transformer, coordinate attention, fruit fly optimization algorithm

## Abstract

**Introduction:**

Glioma is the most common primary malignant tumor of the central nervous system. The mutation status of isocitrate dehydrogenase (IDH) and the methylation status of the O6-methylguanine-DNA methyltransferase (MGMT) promoter are key biomarkers for glioma diagnosis and prognosis. Accurate, non-invasive prediction of these biomarkers using MRI is of significant clinical value.

**Materials and methods:**

We proposed a novel multitask deep learning framework based on Coordinate Attention-EfficientNetV2 (CA-EfficientNetV2) to simultaneously predict IDH mutation and MGMT promoter methylation status based on MRI data. Initially, unlabeled MR images were annotated using K-means clustering to generate pseudolabels, which were subsequently refined using a Vision Transformer (ViT) network to improve labeling accuracy. Then, the Fruit Fly Optimization Algorithm (FOA) was employed to assign optimal weights to the pseudolabeled data. The CA-EfficientNetV2 model, integrated with a coordinate attention mechanism, was constructed. The multitask framework comprised three independent subnetworks: T2-net (based on T2-weighted imaging), T1C-net (based on contrast-enhanced T1-weighted imaging), and TU-net (based on the fusion of T2WI and T1CWI).

**Results:**

The proposed framework demonstrated high performance in predicting both IDH mutation and MGMT promoter methylation status. Among the three subnetworks, TU-net achieved the best results, with accuracies of 0.9598 for IDH and 0.9269 for MGMT, and AUCs of 0.9930 and 0.9584, respectively. Comparative analysis showed that our proposed model outperformed other convolutional neural network (CNN) - based approaches.

**Conclusion:**

The CA-EfficientNetV2-based multitask framework offers a robust, non-invasive method for preoperative prediction of glioma molecular markers. This approach holds strong potential to support clinical decision-making and personalized treatment planning in glioma management.

## 1 Introduction

Glioma is the most common primary malignant brain tumor of the central nervous system in adults, with an annual incidence of approximately 6 per 100,000 persons (1). It can be classified into grades II-IV according to the World Health Organization (WHO). With the introduction of the 2016 revised WHO Classification of Tumors of the Central Nervous System (CNS) (2), molecular biomarkers have become increasingly important for the diagnosis and prognosis of glioma. The latest 2021 WHO classification (3) emphasizes the importance of genetic changes, such as isocitrate dehydrogenase (IDH), in the classification of gliomas. IDH mutant patients have better treatment outcomes and overall survival than the IDH wild patients (4). O6-methylguanine-DNA methyltransferase (MGMT) is also an important prognostic marker for glioma patients (5). MGMT promoter methylation can suppress the activity of MGMT, which is a DNA repair enzyme and can blunt the therapeutic effect of alkylating chemotherapy. Glioma patients with MGMT promoter methylation are more sensitive to chemotherapy and have longer survival time (6). Identification of the status of critical molecular biomarkers for glioma patients at high risk of early progression is critical for personalized treatment planning.

The gold-standard procedure for the identification of glioma molecular biomarkers is a pathological sampling through a brain biopsy or surgery. However, the high risk of complications, high costs and sampling biases due to the inherent heterogeneity of glioma (7) hinder the application of invasive procedures, and support the need for noninvasive and accurate detection of clinically relevant molecular information in glioma patients. Magnetic resonance imaging (MRI) is a routine noninvasive method for detecting brain tumors. In recent years, deep learning networks, especially convolutional neural networks (CNNs), have achieved excellent performances in the medical image processing field, and the features extracted from MR images are related to the gene expression patterns (8–10). Chang et al. (11) used a CNN to independently predict the IDH mutation status, 1p/19q codeletion status and MGMT promoter methylation status, and achieved accuracies of 94, 92 and 83%, respectively. Decuyper et al. (12) designed a 3D U-Net based on preoperative MR images for fully automated segmentations of gliomas, and they split the network into three independent fully connected layers to simultaneously predict tumor grade, IDH mutation and 1p/19q codeletions in gliomas with accuracies of 90, 76 and 75%, respectively. However, these CNNs often require a large amount of labeled data, which leads to a significant waste of clinically available data due to the lack of labels. Recently, the EfficientNet model has gained popularity due to its ability to achieve high accuracies in less time with fewer parameters (13).

In this paper, we propose a multitask deep learning model utilizing glioma MR images, which is based on Coordinate Attention-EfficientNetV2 (CA-EfficientNetV2) to solve the following problems:

- Considering that glioma data often consist of small samples, and that labeled data are difficult to obtain, we propose a pseudolabel annotation algorithm based on K-means clustering and Vision Transformer.
- The pseudolabeling method may decrease the model’s prediction accuracy. Therefore, we propose a weight optimization method for pseudolabeled data based on the fruit fly optimization algorithm to adjust the pseudolabel data weights.
- Most widely used deep neural networks contain a large number of parameters, making them highly complex and computationally inefficient. To solve this problem, the coordinate attention (CA) module is introduced into the EfficientNetV2 lightweight model, and a multitask classification framework based on CA-EfficientNetV2 is proposed.

## 2 Materials and methods

This retrospective study was approved by the ethical review board of the Affiliated Hospital of Xuzhou Medical University. The informed consent requirement was waived.

### 2.1 Data collection

A total of 238 glioma patients from the Affiliated Hospital of Xuzhou Medical University were considered for inclusion. These patients met the following criteria: (i) age greater than or equal to 18 years, (ii) pathologically confirmed glioma (grade II to IV), (iii) preoperative MR images, including axial T2-weighted images (T2WI), T1-weighted contrast-enhanced images (T1CWI) and (iv) no history of surgery or other therapies for brain tumors. All MRI scans were acquired using a 3.0 T GE scanner. The sequence parameters were as follows: T2-weighted imaging (T2WI) with TR = 4,733 ms, TE = 100 ms, field of view (FOV) = 240 mm × 240 mm, and slice thickness = 6.0 mm; T1-weighted contrast-enhanced imaging (T1CWI) with TR = 2,952 ms, TE = 24 ms, FOV = 240 mm × 240 mm, and slice thickness = 6.0 mm.

Among these patients, 71 were diagnosed with WHO grade II gliomas, 55 with grade III, and 112 with grade IV. A total of 44 patients had IDH-mutant tumors, 86 had IDH wild-type, and 108 patients did not have available IDH mutation status. Regarding MGMT promoter methylation, 77 patients had methylated MGMT promoter, 86 had unmethylated MGMT promoter, and 75 lacked MGMT promoter methylation status information. We selected a total of 24 images per patient, including 12 T2WI and 12 T1CWI, acquired from different axial slices at the same time point. For each imaging sequence, 12 representative slices were chosen that best captured the tumor and its surrounding tissue. The selection was guided by anatomical landmarks to ensure comprehensive coverage of the entire tumor regions. The gene mutation status of the dataset was shown in Table 1.

**Table 1:** Gene mutation status of the dataset.

Biomarker	Status	Cases	Image number	Grade		
				II	III	IV
IDH	Mutant	44	1,056	19	15	10
	Wild	86	2,064	12	8	66
	Without labels	108	2,592	40	32	36
MGMT	Methylation	77	1,848	24	16	37
	Nonmethylation	86	2,064	18	19	49
	Without labels	75	1,800	29	20	26

### 2.2 Image preprocessing and data augmentation

All MRI images were preprocessed through a series of standardized steps to ensure uniformity and compatibility with the deep learning framework. First, the MRI images were normalized by rescaling the pixel intensity values to the range of [0, 1] using min-max normalization. After normalization, the images were resampled to standardize their spatial resolution and dimensions. Then, each image was resized to 224 × 224 using bilinear interpolation. Since the original MR images were single-channel grayscale images, and the deep learning models required three-channel RGB inputs, we expanded the single-channel images into three channels. Through the above normalization and resampling process, the MRI images were adjusted to a resolution of 224 × 224 × 3 for model input.

To improve model generalization and address class imbalance, we applied various data augmentation techniques, including horizontal and vertical flipping, as well as random rotations, aimed at increasing the representation of the minority class.

### 2.3 Pseudolabeling based on K-means clustering and vision transformer

The T1CWI and T2WI images were imported into IBEX (14) to for manual delineation of the region of interest (ROI). For each patient, the tumor region was manually delineated on every slice where it was visible. ROI delineation was primarily performed on the T2WI images, with T1CWI images as a reference to ensure complete coverage of the lesion. ROIs were drawn by experienced neuroradiologists under supervision and were subsequently reviewed to ensure consistency and accuracy. The drawn ROIs were saved and exported along with the corresponding image data for radiomic feature extraction based on the gray level co-occurrence matrix (GLCM) and gray level run length matrix (GLRLM). The radiomic features extracted from the MR images constituted an original feature set. To eliminate non-informative features, independent sample t-tests were performed on the labeled data. The selected features were then used to identify useful radiomic features from the unlabeled dataset.

We used K-means clustering algorithm for pseudolabeling. To improve the clustering performance, we specified both the number of clusters (*k* = 2) and biologically informed initial centroids. The two initial centroids were defined as IDH mutant/ MGMT promoter methylated, and IDH wild-type/MGMT promoter unmethylated tumors. These two subtypes were known to differ significantly in terms of prognosis and treatment response in gliomas patients, and their inclusion as initial centroids was intended to guide the clustering process toward meaningful subgroup separation. Through the iterative K-means clustering process, we obtained the mutation status of the unlabeled data by using the similarity between the unlabeled data and the labeled data. In this way, the preliminary pseudolabels were added to the unlabeled data. To evaluate the performance of the pseudolabeling methods, a separate validation subset with known ground truth was used to compute accuracy, precision, and recall of the generated pseudolabels.

**Figure 1:**
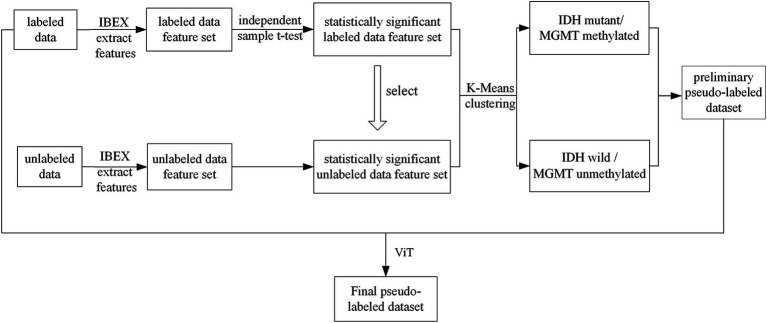
The overall process of pseudolabeling.

**Figure 2:**
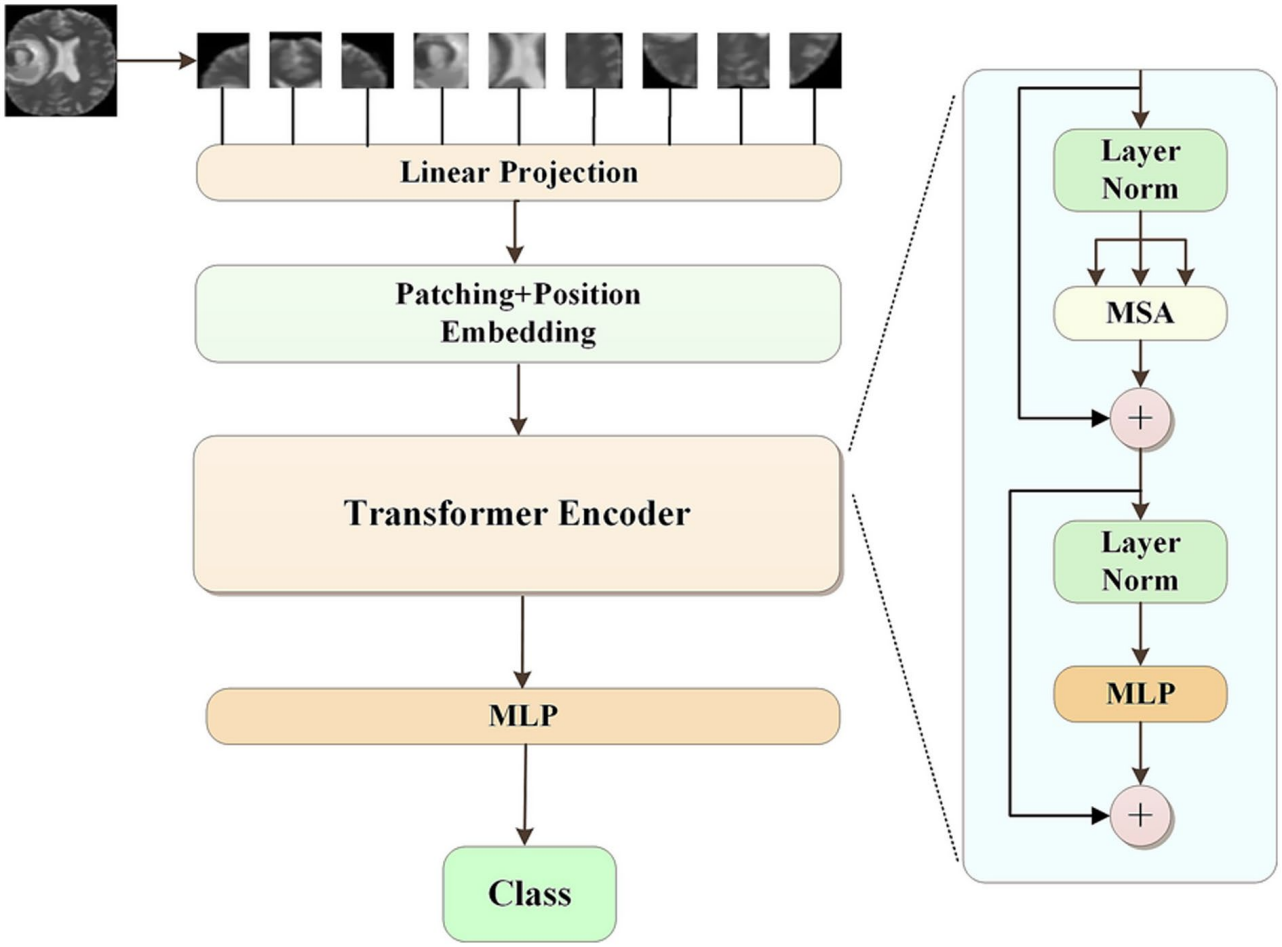
The architecture of vision transformer model.

The pseudolabels obtained from K-means clustering were subsequently refined by a Vision Transformer (ViT) model. The class with the highest predicted probability was selected as the final pseudolabel. The ViT model was consist of several modules. First, the input images were divided into a sequence of flattened 2D patches. These vectorized patches were then projected into a latent 768-dimensional embedding space via a trainable linear projection. To retain spatial information, learnable position embeddings were added to each patch embedding. Additionally, a classification embedding was added to the patch embeddings to retain category information. Next, we fed the patch embeddings along with position and classification embeddings into transformer blocks. Each transformer block consisted of a multihead self-attention (MSA) module and a multilayer perceptron (MLP) module. Finally, we used a MLP block to accomplish the classification task. For the development of our ViT model, the patch size P was set to 16, and the number of transformer blocks was set to 12. The initial learning rate was set to of 1e-4 with a batch size of 32, and the epoch was 100. The model was repeated independently for four times. The overall process of pseudolabeling was shown in Figure 1. The ViT architecture was summarized in Figure 2.

### 2.4 Optimization of pseudolabeled data weights based on the fruit fly optimization algorithm

Since the labeling of pseudolabels might incur some errors, which might adversely affect the model’s accuracy, a pseudolabeled data weight optimization algorithm based on the fruit fly optimization algorithm (FOA) (15, 16) was proposed. We used FOA to find the optimal weights of pseudolabeled data during the training process to improve the accuracy of the model. In the training process, the cross entropy error (CEE) was used as the loss function, as shown in Equation (1):

(1) $$L_{c}=-[y_{i}\log(f(x_{i})+(1-y_{i})\log(1-f(x_{i}))]$$

Assume that the glioma image dataset has a total of N data points, including N_k_ labeled data, $X_{k}=\{(x_{1},y_{1}),(x_{2},y_{2}),\ldots ,(x_{N_{k}},y_{N_{k}})\}$ , and (N-Nk) pseudolabeled data, $X_{l}=\{(x_{N_{k+1}},y_{N_{k+1}}),(x_{N_{k+2}},y_{N_{k+2}}),\ldots ,(x_{N},y_{N})\}$ ; then, the loss function during the training period was expressed as shown in Equation (2):

(2) $$L=\sum_{i=1}^{N}L_{c}(f(x_{i}),y_{i})$$

The loss function was adjusted by fixing the weight of the real labeled data to 1 and applying different weights to the pseudolabeled data. Since the labeled data and the pseudolabeled data had different degrees of importance, the loss function could be re-expressed as shown in Equation (3):

(3) $$L\prime =\sum_{i=1}^{N_{k}}L_{c}(f(x_{i}),y_{i})+\sum_{i=N_{k}+1}^{N}L_{c}(\mathrm{\alpha f}(x_{i}),y_{i})$$

where *α* was the weight applied to the pseudolabeled data.

The optimization algorithm of the pseudolabeled data weights based on FOA was shown in Algorithm 1, where popsize was the population size of the fruit flies, Maxgen was the maximum number of iterations, R was the Drosophila flight radius, D was the number of optimization variables, bestSmell was the optimal flavor concentration, and *α* was the pseudolabel data weight.

**ALGORITHM 1:**
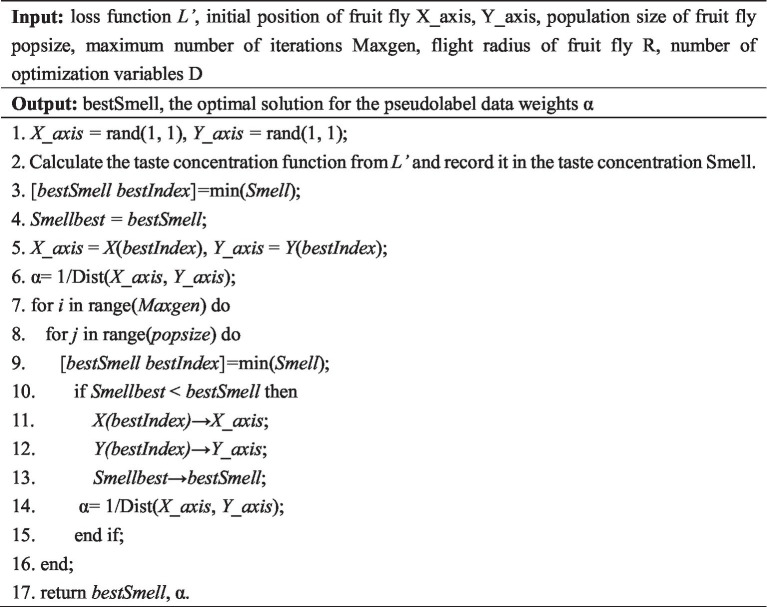
A pseudolabel data weight optimization algorithm based on the fruit fly optimization algorithm.

### 2.5 A multitask classification framework based on CA-EfficientNetV2

#### 2.5.1 CA-EfficientNetV2

In this study, EfficientNetV2-S (17) was used as the backbone network. Considering the computational cost and training speed, the network structure was shown in Table 2.

**Table 2:** EfficientNetV2-S network structure.

Stage	Operator	Stride	Channels	Layers
0	Conv3 × 3	2	24	1
1	Fused-MBConv1, k3 × 3	1	24	2
2	Fused-MBConv4, k3 × 3	2	48	4
3	Fused-MBConv4, k3 × 3	2	64	4
4	MBConv4, k3 × 3, SE0.25	2	128	6
5	MBConv6, k3 × 3, SE0.25	1	160	9
6	MBConv6, k3 × 3, SE0.25	2	256	15
7	Conv1 × 1 and Pooling and FC	-	1,280	1

The Fused-MBConv (Fused Mobile Inverted Bottleneck Convolution) was a simplified and efficient variant of the standard MBConv block, introduced in EfficientNetV2 (17). Unlike MBConv, which used a sequence of expansion (1 × 1 convolution), depthwise convolution (3 × 3), and projection (1 × 1 convolution), the Fused-MBConv eliminated the depthwise convolution and merged the expansion and spatial filtering into a single 3 × 3 regular convolution. This design reduced memory access cost and improved computational efficiency, especially in the early layers where input resolution was high. In our study, we replaced the squeeze and excitation (SE) attention block contained in the MBConv module in EfficientNetV2 with a coordinate attention (CA) block to optimize the network structure. CA utilizes two one-dimensional global pooling operations to aggregate the vertical and horizontal input features into two independent direction-aware feature maps, and then encodes the two feature maps with embedded direction-specific information into two attention maps. Each attention map captures the long-range dependencies of the input feature maps along one spatial direction. Therefore, positional information could be saved in the generated attention map, and the attention maps of two independent directions were applied to the input feature map by multiplication to emphasize the representation of interest. In our study, global pooling was transformed into an encoding operation of two 1D vectors. For input X, the pooling kernels (H, 1) and (1, W) were used to encode the horizontal and vertical features, and the output of the c-th dimensional feature was:

(4) $$z_{c}^{h}(h)=\frac{1}{W}\sum_{0\leq i\leq W}x_{c}(h,i)$$

(5) $$z_{c}^{w}(w)=\frac{1}{H}\sum_{0\leq j\leq H}x_{c}(j,w)$$

**Figure 3:**
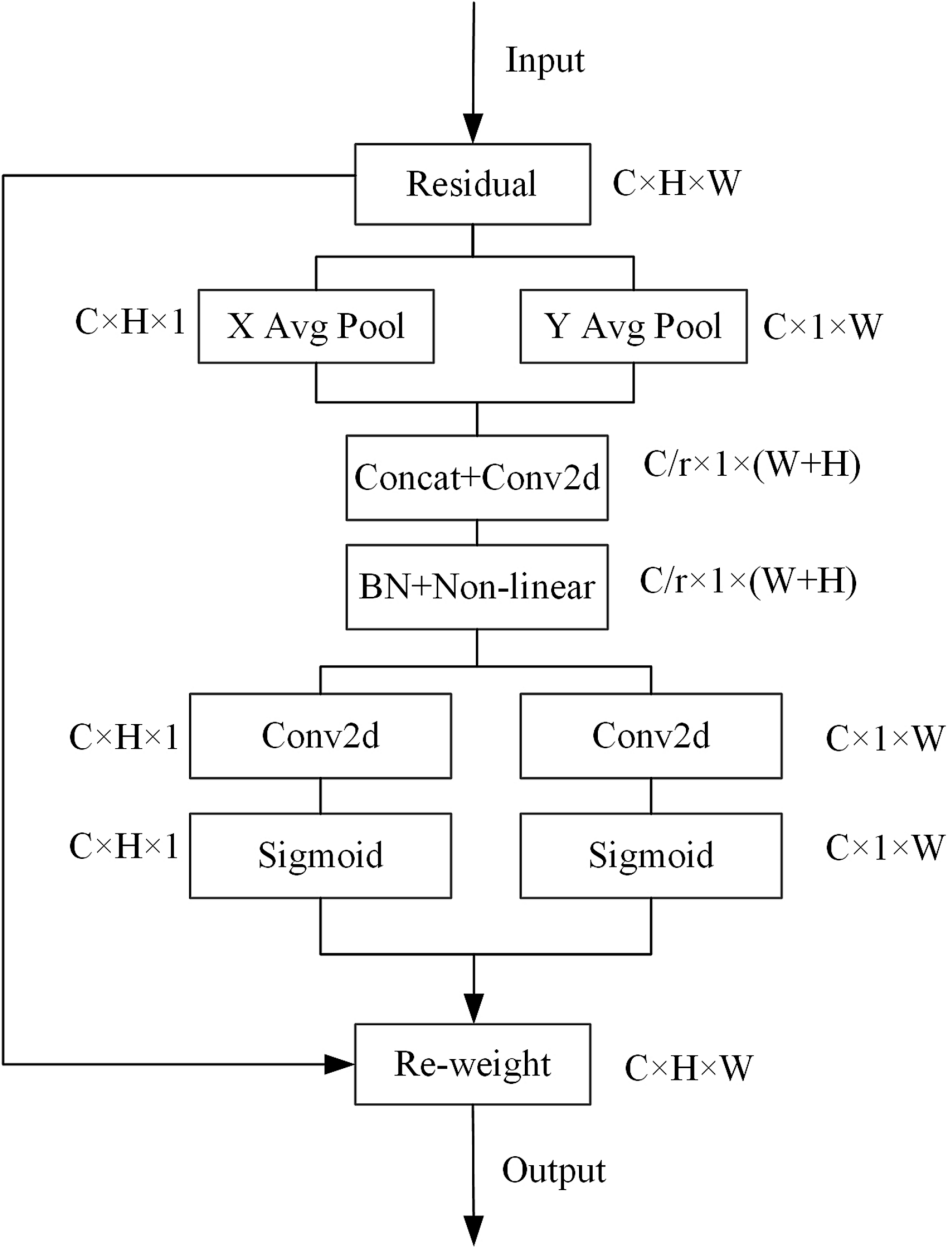
CA module structure.

Equations 4 and 5 integrate features from different directions and output a pair of directionally knowable feature maps. We used a reduction ratio 𝑟 = 32, a kernel size of 1 for the shared convolutional layer, and ReLU and Sigmoid activations for intermediate and final mappings, respectively. The specific structure of the CA module was shown in Figure 3.

#### 2.5.2 A multitask classification framework based on CA-EfficientNetV2

**Figure 4:**
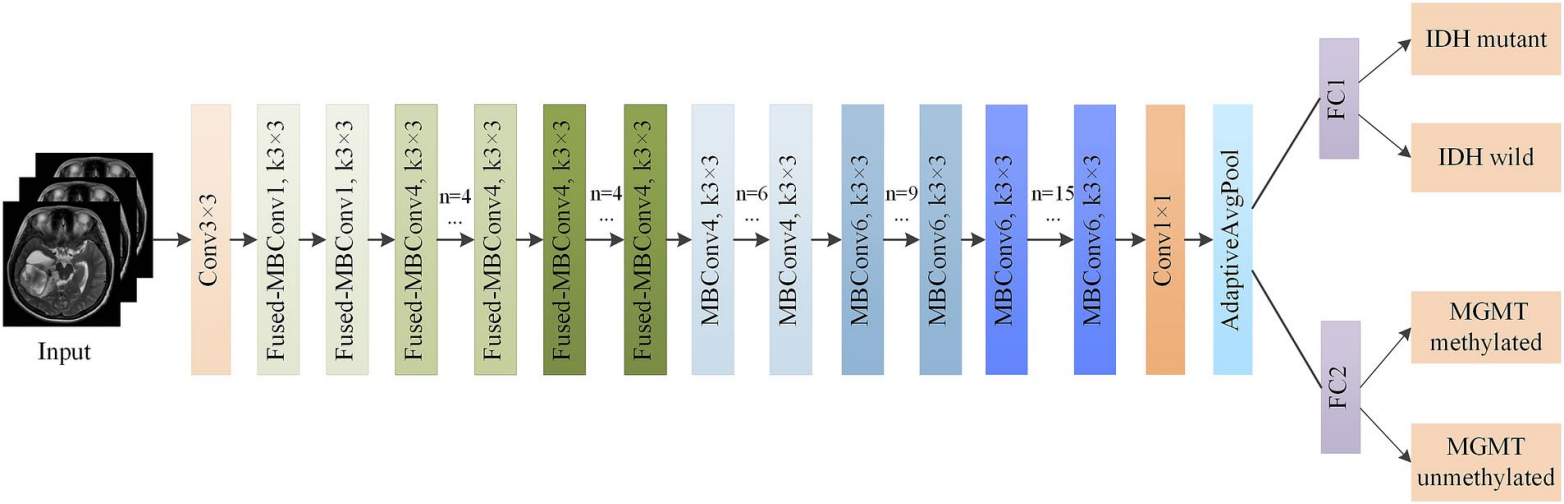
Structure of multitask classification model based on CA-EfficientNetV2.

As shown in Figure 4, the output of the CA-EfficientNetV2 network was split into two independent fully connected (FC) layers to predict both IDH mutation and MGMT methylation statuses. We applied the multitask model to construct independent networks based on T2WI, T1CWI and T2 + T1CWI (T2-net, T1C-net and TU-net).

To prevent data leakage, a patient-level train-test split was performed. All images from each patient were exclusively assigned to either the training set or the test set to avoid any potential data leakage. The dataset was randomly partitioned, with 80% of the patients used for training and 20% for testing. We used PyCharm as the development environment, PyTorch as the deep learning framework, and Python as the programming language. The training set and the testing set were divided at a ratio of 8:2. The model was trained using the Adam optimizer with an initial learning rate of 1e-4. A piecewise constant decay strategy was employed, where the learning rate was multiplied by a decay factor of 0.1 every 30 epochs. This approach facilitated rapid convergence in the early stage of training while mitigating oscillations in the later stage, thereby enhancing overall optimization performance. The batch size was set to 32, and the model was trained for 100 epochs.

### 2.6 The CNN-based network development

Five of the widely used CNN models, including Xception, ResNet-50, DenseNet-121, MobileNet-V2, EfficientNet-B0 models, were selected for the comparison with our proposed model in terms of prediction performance. All baseline models were initialized with ImageNet-pretrained weights and subsequently fine-tuned on our dataset. To ensure a fair comparison, all models were trained using the same protocol, including the Adam optimizer, a learning rate of 1e-4, and a batch size of 32. Furthermore, identical data preprocessing and image augmentation procedures were applied across all models.

### 2.7 Statistical analysis

To evaluate the model’s performance, the metrics accuracy, precision, recall and F1-Score were calculated based on the equations (TP: true positive; TN: true negative; FP: false positive; FN: false negative).

$$\text{Accuracy}=\frac{\mathrm{TP}+\mathrm{TN}}{\mathrm{TP}+\mathrm{TN}+\mathrm{FP}+\mathrm{FN}}$$

$$\text{Precision}=\frac{\mathrm{TP}}{\mathrm{TP}+\mathrm{FP}}$$

$$\text{Recall}=\frac{\mathrm{TP}}{\mathrm{TP}+\mathrm{FN}}$$

$$F_{1}-\text{Score}=\frac{2\mathrm{TP}}{2\mathrm{TP}+\mathrm{FP}+\mathrm{FN}}$$

The receiver operating characteristic (ROC) curve was plotted, and the area under the ROC curve (AUC) was calculated to measure the classification accuracy. The parameters were calculated in PyCharm with the programming language Python (version 3.10; Wilmington, DE, USA).^1^

## 3 Results

### 3.1 Comparisons of the pseudolabeling algorithms

**Figure 5:**
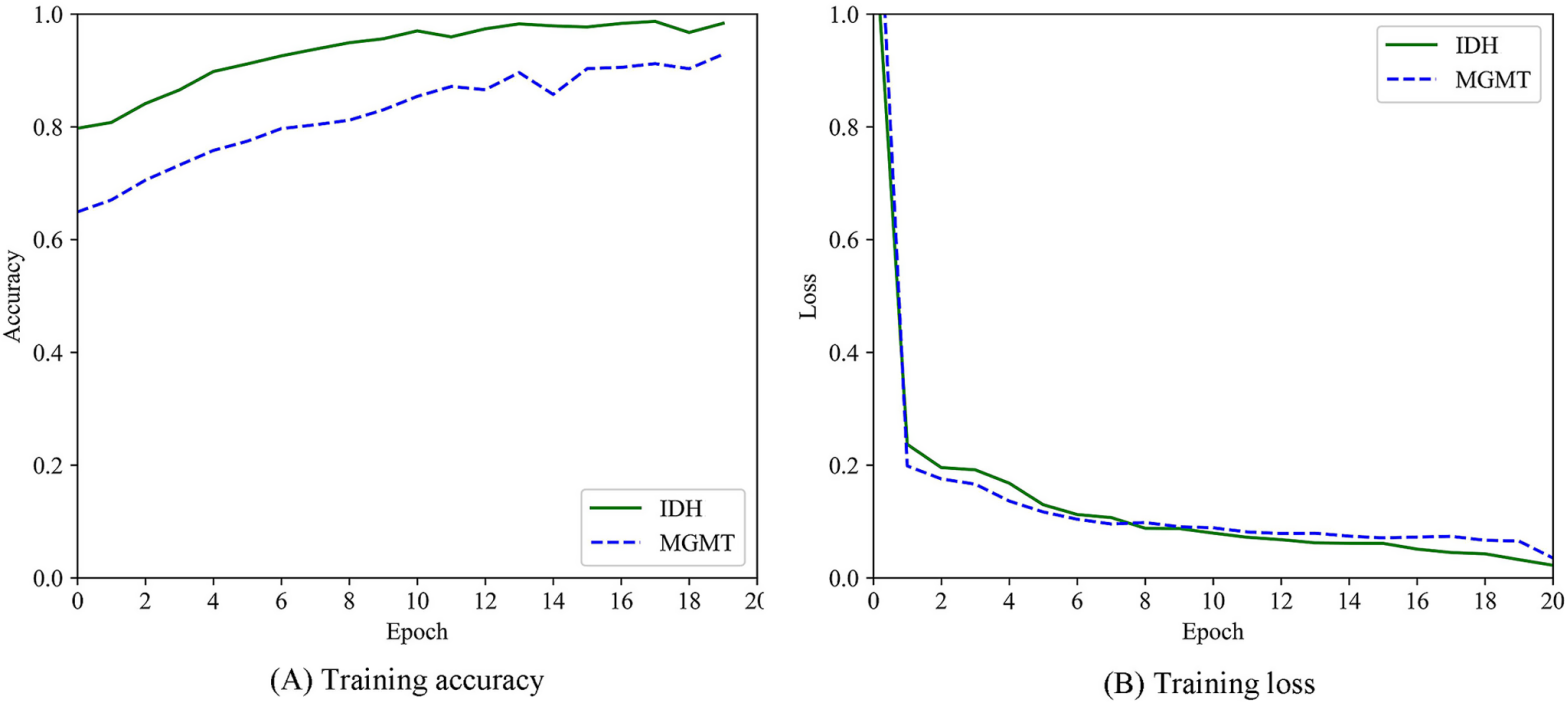
Training process of ViT model. **(A)** Training accuracy. **(B)** Training loss.

A total of 363 radiomics features were extracted in this experiment, including 330 features derived from the GLCM and 33 features derived from the GLRLM. Through an independent sample *t*-test, 94 statistically significant features were retained. Based on these 94 features, the labeled data and the unlabeled data were clustered together. Then, the ViT model was used to correct the pseudolabels obtained from the clustering. The training process was shown in Figure 5. The accuracy of the 108 pseudolabels for the prediction of the IDH mutation status was 83.33%, and the accuracy of the 75 pseudolabels for the prediction of the MGMT promptor methylation status was 81.93%. The accuracy comparisons among the different pseudolabeling algorithms were shown in Table 3.

**Table 3:** Accuracy of pseudolabeling algorithms (K-means and ViT).

Parameter	Method	IDH	MGMT
Accuracy	Attribute reduction	0.7812	0.7684
	K-means clustering	0.8077	0.7968
	K-means clustering + ViT	0.8333	0.8193
AUC	Attribute reduction	0.8429	0.7928
	K-means clustering	0.8841	0.8542
	K-means clustering + ViT	0.9092	0.9001
Time (s)	Attribute reduction	657	669
	K-means clustering	368	375
	K-means clustering + ViT	425	429

Accuracy was computed using a held-out labeled validation subset, which was used solely to evaluate pseudolabeling quality.

### 3.2 Optimization result of pseudolabel data weight based on the fruit fly optimization algorithm

**Figure 6:**
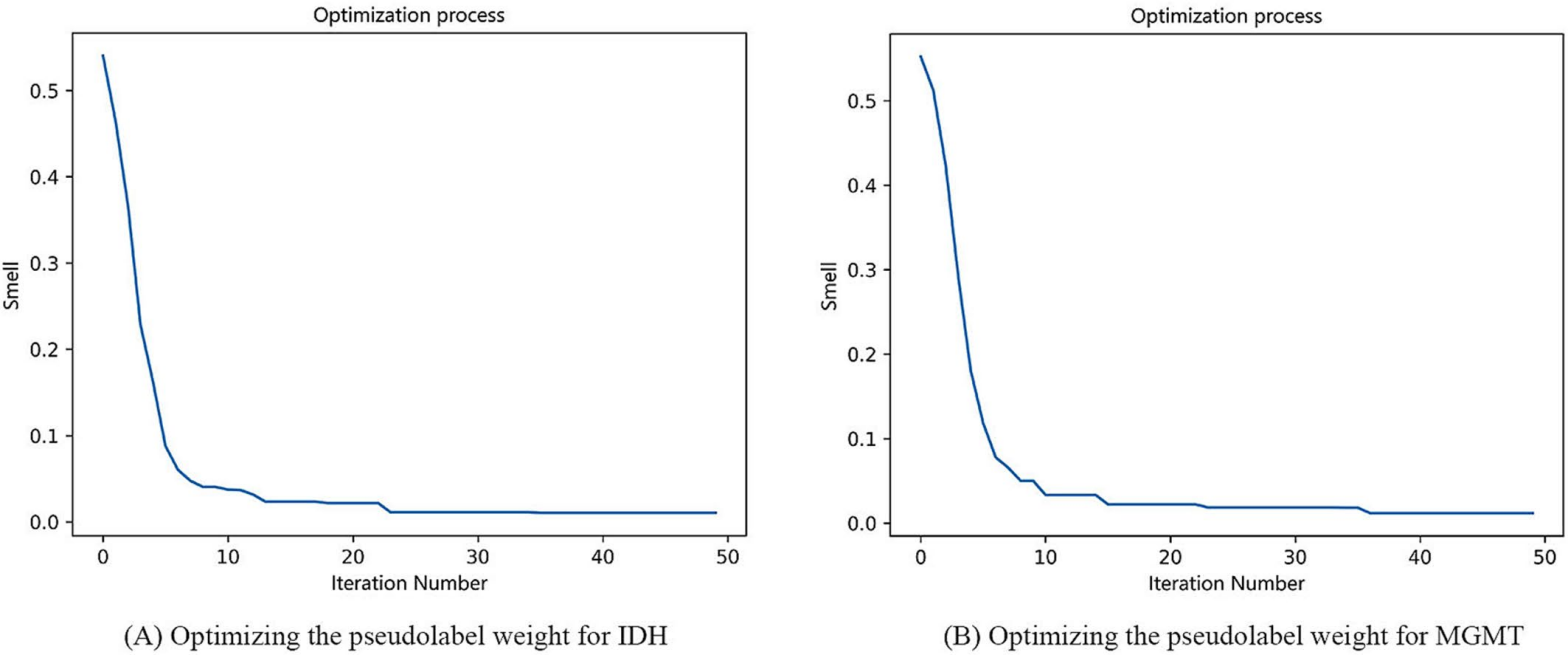
Process of optimizing the pseudolabel weight for IDH mutation **(A)** and MGMT methylation **(B)**.

In our study, the weight of the labeled data was fixed at 1, and the weight of the pseudolabeled data was adjusted by the FOA. In the optimization process using the FOA, the population size was set to 5, the maximum number of iterations (MaxGen) was set to 50, the radius (R) was set to 1, and the number of optimization variables (D) was set to 1. When the flavor concentration reached the optimal value, the optimization was over. The process of optimizing the pseudolabel weight for IDH mutation and MGMT methylation was shown in Figure 6. For IDH mutation, when the final taste concentration was 0.0104, and the weight of the pseudolabel data reached the optimal value of 0.17. For MGMT methylation, when the final taste concentration was 0.0118, the weight of the pseudolabel data reached the optimal value of 0.12.

### 3.3 Performance of multitask classification networks based on CA-EfficientNetV2

**Figure 7:**
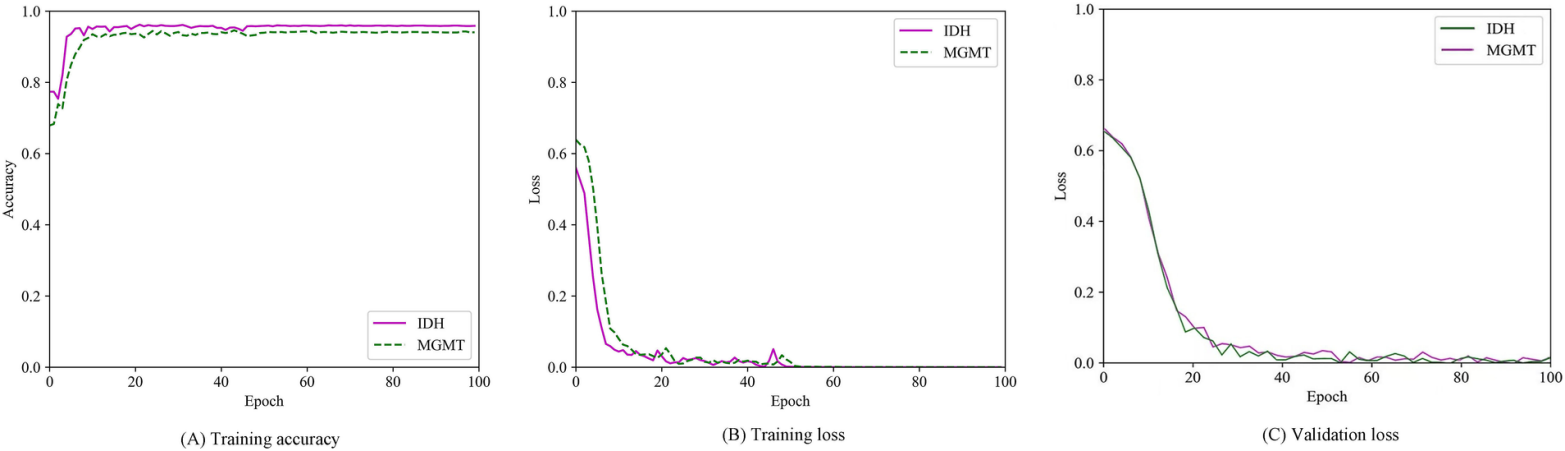
Training accuracy curve **(A)**, training loss curves **(B)** and validation loss curve **(C)** over epochs.

**Figure 8:**
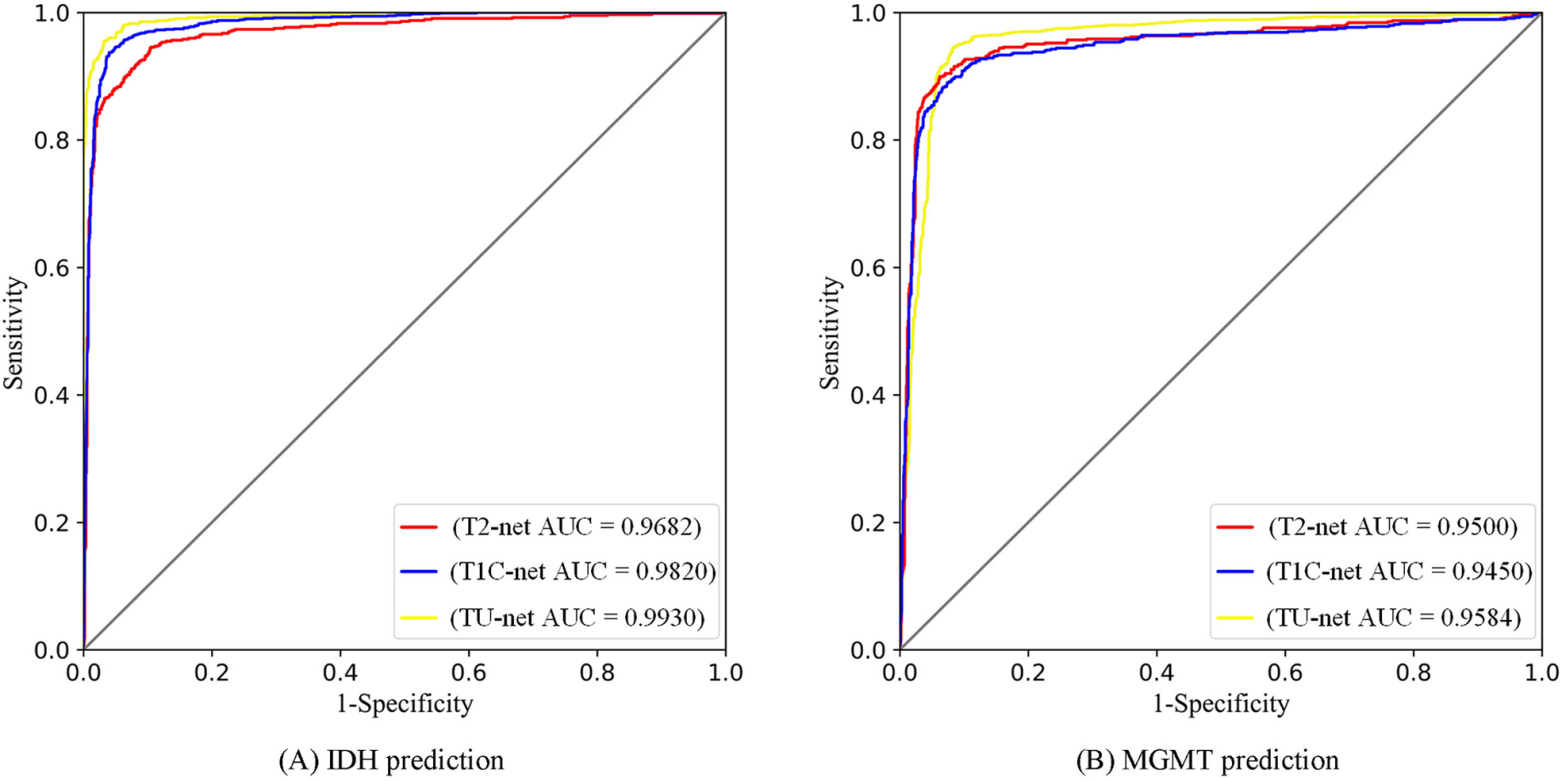
ROC curves of multitask classification networks (including T2-net, T1C-net and TU-net) for the prediction of IDH **(A)** and MGMT **(B)** statuses.

The multitask classification model converged after 100 iterations, and the training and validation processes were depicted in Figure 7. We compared three independent networks (T2-net, T1C-net and TU-net) based on the proposed multitask classification model. Performance comparisons of the three networks were shown in Table 4. The three networks showed high accuracy and AUCs in predicting both IDH mutation and MGMT promoter methylation. Compared with T2-net and T1C-net, TU-net achieved the best performance with the highest accuracy and AUC. The ROC curves of the multitask classification networks (including T2-net, T1C-net and TU-net) were shown in Figure 8.

**Table 4:** The prediction performance of the three networks based on CA-EfficientNetV2.

Parameter	Biomarker	T2-net	T1C-net	TU-net
Accuracy	IDH	0.9231 (0.8929, 0.9533)	0.9470 (0.9238, 0.9702)	0.9598 (0.9409, 0.9787)
	MGMT	0.9143 (0.8786, 0.9500)	0.9132 (0.8773, 0.9491)	0.9269 (0.8949, 0.9589)
AUC	IDH	0.9682 (0.9492, 0.9872)	0.9820 (0.9705, 0.9935)	0.9930 (0.9877, 0.9983)
	MGMT	0.9500 (0.9232, 0.9768)	0.9450 (0.9168, 0.9732)	0.9584 (0.9364, 0.9804)
Precision	IDH	0.9201	0.9472	0.9593
	MGMT	0.9161	0.9076	0.9268
Recall	IDH	0.9153	0.9468	0.9538
	MGMT	0.9180	0.9073	0.9270
F1-score	IDH	0.9176	0.9469	0.9564
	MGMT	0.9225	0.9075	0.9269

Values in parentheses represent 95% confidence intervals.

### 3.4 Ablation experiments and comparisons with CNN-based models

To validate the effectiveness of the proposed model, a series of ablation experiments were performed. Table 5 showed that the accuracy, precision, recall, F1-score and AUC of the model decreased without using the CA module to replace the SE attention block in EfficientNetV2-S, without using the FOA to optimize the pseudolabeled data weights, and without using the pseudolabeled data. These results demonstrated that each component - the CA module, the FOA, and the pseudo-labeling strategy - played a significant role in enhancing the model’s predictive performance.

**Table 5:** Ablation study of key components in the CA-EfficientNetV2 model for IDH mutation and MGMT promoter methylation status prediction.

Parameter	Biomarker	Full model	w/o CA module	w/o FOA	w/o Pseudolabel data	w/ Backbone network
Accuracy	IDH	0.9587	0.9474	0.9406	0.9399	0.9192
	MGMT	0.9406	0.9249	0.9188	0.9115	0.9013
Precision	IDH	0.9598	0.9499	0.9418	0.9307	0.9273
	MGMT	0.9269	0.9265	0.9208	0.9190	0.9086
Recall	IDH	0.9593	0.9217	0.9417	0.9206	0.9272
	MGMT	0.9268	0.9264	0.9158	0.9184	0.9071
F1-Score	IDH	0.9538	0.9178	0.9420	0.9099	0.9275
	MGMT	0.9270	0.9266	0.9129	0.9058	0.8941
AUC	IDH	0.9564	0.9197	0.9418	0.9150	0.9273
	MGMT	0.9269	0.9265	0.9143	0.9114	0.8999

w/o = without; w/ = with.

CNN-based models, including Xception, ResNet-50, DenseNet-121, Mobile-NetV2 and EfficientNet-B0 were compared with our CA-EfficientNetV2 model in terms of prediction performance. Table 6 showed that our CA-EfficientNetV2 model was superior to all baseline CNN models in terms of accuracy, precision, recall, F1-score and AUC.

**Table 6:** Comparison of our CA-EfficientNetV2 model with CNN-based models in terms of prediction performance.

Parameter	Biomarker	Xception	ResNet-50	DenseNet-121	MobileNet-V2	EfficientNet-B0	CA-EfficientNetV2
Accuracy	IDH	0.7831	0.6667	0.8692	0.7576	0.7624	**0.9598**
	MGMT	0.8421	0.7751	0.7416	0.7544	0.7911	**0.9269**
Precision	IDH	0.9000	0.5239	0.8531	0.8585	0.7821	**0.9593**
	MGMT	0.8464	0.7802	0.7203	0.7763	0.8078	**0.9268**
Recall	IDH	0.4655	0.8678	0.7792	0.3991	0.5148	**0.9538**
	MGMT	0.8820	0.8587	0.9033	0.8104	0.8239	**0.9270**
F1-Score	IDH	0.6136	0.6534	0.8145	0.5449	0.6209	**0.9564**
	MGMT	0.8638	0.8176	0.8015	0.7930	0.8158	**0.9269**
AUC	IDH	0.9042	0.8399	0.9385	0.8054	0.8181	**0.9930**
	MGMT	0.9299	0.8541	0.9225	0.8149	0.8602	**0.9584**
Time (s)	IDH	658	754	674	545	524	**385**
	MGMT	660	759	680	552	528	**385**

Bold values indicate the best performance among the compared models.

## 4 Discussion

In this study, we proposed a multitask MR-based deep learning framework based on CA-EfficientNetV2 for the automatic prediction of molecular biomarkers in glioma patients. In the absence of a large amount of labeled clinical data, we proposed a pseudolabeling method to add pseudolabels to the data and improved upon the lightweight model EfficientNetV2. The results showed that our proposed model could accurately predict IDH mutation and MGMT promopter methylation simultaneously in glioma patients. Compared with other CNN-based models, our proposed CA-EfficientNetV2 model outperformed these classic CNN models on the same dataset.

The training of deep neural network models usually requires a large amount of labeled data to achieve the desired results. Due to the limited amount of labeled data used in clinical practices, we performed data augmentation by labeling the unlabeled glioma data with pseudolabeling algorithms. Pseudolabeling (18) is a method of adding labels to unlabeled data based on labeled data, which improves the robustness of the model and avoids overfitting. In this study, we proposed a pseudolabeling algorithm based on K-means clustering and Vision Transformer to improve the performance of the algorithm. The conventional K-means clustering algorithm randomly selects the initial centroid, and the clustering effect is affected by the selection of the initial centroid. Here, we specified two initial centroids of mass, the IDH mutant type/MGMT promoter methylated samples and IDH wild type/MGMT promoter unmethylated samples, because of the strong association between IDH mutation and MGMT promoter methylation in gliomas (19, 20). Recently, Vision Transformer (ViT) was introduced in the field of medical image analysis (21, 22); it recruits self-attention mechanisms in image patches and has demonstrated promising results, even fully replacing pure CNN models. After clustering by the K-means algorithm, the ViT model was introduced to speed up the convergence of the model. As shown in Table 3, compared with the attribute reduction method or K-means clustering method alone, K-means clustering combined with the ViT method, achieved the highest accuracy in the shortest time. Although the computational requirements of ViT might limit its applicability in resource-constrained environments, due to the focus of this study, we did not explore model optimizations or alternative architectures such as the Swin Transformer. Future work will focus on investigating these optimization strategies to improve the efficiency and practicality of our model in clinical applications.

In our study, we employed the Fruit Fly Optimization Algorithm (FOA) to optimize the weights of the pseudo-labeled data, thereby enhancing the accuracy of the model. The computational efficiency and practical applicability of FOA were critical considerations in the context of medical image processing. FOA is a lightweight and effective metaheuristic algorithm with limited number of parameters (15). In our study, the FOA parameters were deliberately set to conservative values (population size = 5, MaxGen = 50, and D = 1) to ensure rapid convergence without compromising accuracy. The algorithm demonstrated consistent and stable performance, highlighting its robustness. Moreover, FOA’s low computational overhead makes it feasible for deployment in clinical settings (16). Many medical image processing applications, such as computer-aided diagnosis or image segmentation, require quick response times. The simplicity and speed of FOA enable real-time execution on standard clinical hardware, without the need for high-performance computing infrastructure.

EfficientNet (13) is a neural search network proposed by a Google team in 2019. By using Neural Architecture Search (NAS), it searches for a rational configuration of network depth, width and resolution, thus obtaining better performances than CNNs. In 2021, the Google team released EfficientNetV2 (17), which introduced the Fused-MBConv module to the search space, they also proposed a progressive learning strategy that could adjust the regularization factor based on the size of the image, thus accelerating the training process and improving the model accuracy compared with EfficientNet. In our study, we improved the EfficientNetV2 model by replacing the original SE attention mechanism with coordinate attention and adjusting the weights of the pseudolabeled data via the fruit fly optimization algorithm. To highlight the performance capabilities of our proposed CA-EfficientNetV2 model, we performed ablation experiments and compared it with other classical CNN models. The CA-EfficientNetV2 model yielded a better performance than these CNN-based models in shorter time. There are several potential reasons for this. First, CNN models typically contain a large number of parameters, which makes it difficult for them to effectively capture localized lesion features, especially when trained on a relatively small dataset. Second, CA embeds positional information into channel attention and can capture both long-range dependencies and positional information along two spatial directions (23), while SE uses global pooling to compress the global information into a single feature vector and has difficulty in retaining important positional information (24). Therefore, by using CA block, the proposed model can locate targets more accurately and extract more representative features from the MR images. Third, we use a fruit fly optimization algorithm to adjust the weights of the pseudolabeled data to reduce the impact of inaccurate pseudolabeling on model accuracy. In addition, thanks to the training-aware neural architecture search (NAS) and compound scaling strategy, our proposed model outperforms other CNN models in inference speed, making it a promising candidate for future clinical applications.

In this study, we employed a multitask classification framework based on a hard parameter sharing mechanism for the simultaneous predictions of IDH mutation and MGMT methylation. Genomic analysis, laboratory studies and case series have demonstrated a strong association between IDH mutation and MGMT methylation. The molecular basis may be that IDH mutation lead to the accumulation of 2-hydroxyglutarate (2-HG), which inhibits the activity of histone and DNA demethylases, resulting in DNA hypermethylation and histones that drive the disease phenotype (25). By sharing most of the parameters among the same hidden layers between multiple tasks, hard parameter sharing greatly reduces the risk of overfitting and improves the performance of the model when dealing with tasks with strong correlation (26), which also explains the high accuracy of the model.

In our study, we developed three networks using different MR image sequences, namely, T2-net, T1C-net and TU-net. Among these networks, TU-net outperformed the other two networks. In the field of radiogenomics, specific imaging features are correlated with glioma molecular markers to identify their imaging phenotypes, assuming that the tumor molecular differences and biological behavior are mirrored in the imaging features (27, 28). Previous studies have shown that a greater percentage of nonenhancing tumors, frontal lobe localization, a larger tumor size and the presence of cysts and satellites are correlated with IDH mutation (29); limited peritumoral edema and mixed nodular enhancement are potentially indicative of MGMT methylation (29, 30). These imaging features are mainly obtained from T2WI and T1CWI, which indicates that the features extracted from T2WI and T1CWI provide additional key information to predict tumor phenotypes.

Previous studies by Choi et al. (8) and Bangalore Yogananda et al. (10) employed single-task deep learning models focused exclusively on predicting IDH mutation status, highlighting the robustness of their models across large-scale, multi-institutional datasets. In contrast, our study proposed a multitask deep learning framework capable of simultaneously predicting two critical molecular biomarkers - IDH mutation and MGMT promoter methylation - and achieved superior accuracy and AUC in both tasks. This highlighted the model’s enhanced clinical utility and diagnostic value. Furthermore, our framework incorporated a novel data augmentation strategy, which involved generating and refining pseudo-labels through K-means clustering and Vision Transformer-based correction, along with a weighting mechanism based on the Fruit Fly Optimization Algorithm (FOA). This approach effectively increased the quantity and quality of training data, making the model particularly well-suited for real-world scenarios involving limited or weakly annotated datasets. Furthermore, we measured the average time required for image preprocessing and model inference, with the total processing time per case being approximately 7 min, suggesting the model’s potential feasibility for real-time clinical application.

There are several limitations to our study. First, the current sample size is relatively limited. To confirm the robustness and generalizability of our model, validation on larger, multicenter datasets is necessary. Future research should incorporate multiple external datasets from diverse institutions to more comprehensively evaluate the model’s performance across varied imaging protocols and patient populations. Second, the proposed model relies solely on structural MR images. We chose to focus on structural MRI images for simplicity and to avoid the additional complexity that would arise from integrating functional MRI or clinical data. However, we recognize the potential benefits of incorporating such data. Functional MRI (fMRI) can provide valuable information on brain activity and connectivity, which may be linked to tumor characteristics and prognosis. Similarly, clinical data (e.g., age, sex and tumor location) can offer important insights into the patient’s response to treatment and disease progression. In future work, we plan to integrate these additional data sources to explore their potential in improving prediction accuracy.

## 5 Conclusion

We proposed a multitask CA-EfficientNetV2 model based on MR imaging for the simultaneous prediction of IDH mutation and MGMT promoter methylation in gliomas. Firstly, in order to increase the available data, the pseudolabels were added to the unlabeled MR images using the K-means clustering algorithm and Vision Transformer network. Secondly, the fruit fly optimization algorithm was used to assign optimal weights to the pseudo-labeled data to improve the accuracy of the model. Finally, CA block combined with EfficientNetV2 model was built to simultaneously predict the IDH mutation and MGMT promoter methylation status of glioma. The proposed model can accurately predict glioma gene mutation statuses, and is superior to other CNN models.

## Data Availability

The raw data supporting the conclusions of this article will be made available by the authors, without undue reservation.

## Footnotes

^1^ https://www.python.org/
